# Depletion of all platelet integrins impacts hemostasis, thrombosis, and tumor metastasis

**DOI:** 10.1016/j.isci.2025.113250

**Published:** 2025-07-31

**Authors:** Emily Janus-Bell, Cristina Liboni, Alexandra Yakusheva, Vincent Mittelheisser, Clarisse Mouriaux, Catherine Bourdon, Louis Bochler, Vincent Hyenne, Maria Garcia-Leon, Olivier Lefebvre, Jacky G. Goetz, Pierre H. Mangin

**Affiliations:** 1 Université de Strasbourg, INSERM, EFS Grand Est, BPPS UMR-S1255, FMTS, 67065 Strasbourg, France; 2 Tumor Biomechanics, INSERM UMR_S1109, 67000 Strasbourg, France; 3 Université de Strasbourg, Strasbourg, France; 4 Fédération de Médecine Translationnelle de Strasbourg (FMTS), Strasbourg, France; 5 Equipe Labellisée Ligue Contre le Cancer, Lyon, France; 6 CNRS, SNC5055, Strasbourg, France

**Keywords:** Cell biology, Cancer

## Abstract

Platelet integrins, together with other platelet receptors, are known to control hemostasis and thrombosis but also metastatic progression. However, the impact of their exclusive but combined deficiency has never been tested in these processes. In a PF4Cre-β1^−/−^/β3^−/−^ mouse strain, we found that platelets were exclusively depleted of all integrins. While they displayed impaired binding to fibrinogen and annexin V, P-selectin exposure was normal. Platelet adhesion was abrogated on immobilized fibrinogen and fibrillar fibronectin under shear flow. PF4Cre-β1^−/−^/β3^−/−^ mice presented an increased bleeding time and a profound defect in experimental models of arterial thrombosis. Platelet adhesion to tumor cells was also reduced, with a pronounced effect on tumor growth and metastatic burden in a model of triple negative breast cancer. Overall, these results confirm the central role of platelet integrins in hemostasis and thrombosis, and highlight their contribution to tumor growth and metastasis formation.

## Highlights

• Platelets of PF4Cre-β1^−/−^/β3^−/−^ mice are exclusively depleted of all integrins
• Binding of integrin-depleted platelets fibrinogen and tumor cells is reduced
• PF4Cre-β1^−/−^/β3^−/−^ mice show increased bleeding time and thrombosis defects
• PF4Cre-β1^−/−^/β3^−/−^ mice display reduced mammary tumor growth and metastasis

## Graphical abstract

**Figure:**
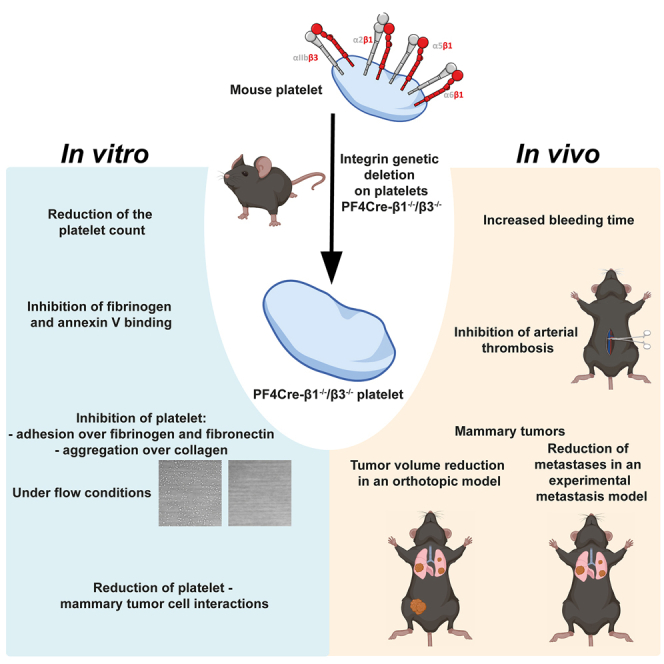

## Introduction

Platelets are small anucleated circulating blood cells, which play a central role in hemostasis. At site of vessel injury, they adhere, activate, and aggregate to form a platelet plug that stops bleeding. Platelet adhesion at site of injury is molecularly initiated through interaction of the glycoprotein Ib-IX-V (GPIb-IX-V) complex with subendothelial von Willebrand factor (vWF).^1^^,^^2^ GPIb-vWF bond formation is crucial for platelet attachment at elevated wall shear rates. Under lower shear conditions, platelet β1 and β3 integrins ensure the recruitment of circulating platelets themselves.^1^^,^^2^ In addition, platelet integrins are particularly important to stabilize adhesion of the cells to extracellular matrix (ECM) proteins. Once platelets have stably adhered, their activation is mostly initiated through interaction of glycoprotein VI (GPVI) with collagen.^3^^,^^4^ This induces the conformational change of integrin αIIbβ3 into a state with high affinity for circulating fibrinogen,^5^ inducing platelet aggregation and the formation of a platelet plug sealing the breach.^6^ Platelets are also key factor in arterial thrombosis, which is initiated upon rupture of an atherosclerotic plaque in a diseased artery. This pathological process leads to the formation of a thrombus and thereby to life-threatening ischemic pathologies such as myocardial infarction or ischemic stroke.^7^

Integrins are heterodimeric transmembrane receptors composed of an α and a β chain. Platelets express at their surface three β1 integrins, α2β1, α5β1, and α6β1, interacting with collagen, fibronectin, and laminins, respectively,^8^^,^^9^^,^^10^^,^^11^ and two β3 integrins, αvβ3 and αIIbβ3, interacting with vitronectin and fibrinogen.^12^^,^^13^ The role of β1 integrins is limited to platelet adhesion and activation on ECM proteins, with no major effect on hemostasis as evidenced by a normal tail bleeding time in platelet α2-, α5-, and α6-deficient mice.^14^^,^^15^^,^^16^ Furthermore, while no patient with a deficiency in one of the platelet β1 integrins has been identified, a patient with depletion of the α2 chain presented mild bleeding during childhood but then a decreased risk of bleeding in adulthood.^17^ Integrins α2β1 and α6β1, but not α5β1, are implicated in thrombosis since their genetic depletion in mice led to reduction of thrombosis in several experimental models.^15^^,^^16^^,^^18^^,^^19^ Concerning platelet β3 integrins, the most abundant is αIIbβ3, which plays a central role in platelet aggregation through its interaction with circulating fibrinogen. The importance of αIIbβ3 in hemostasis is evidenced by Glanzmann’s thrombasthenia, a hemorrhagic disease caused by its absence or non-functionality.^20^ This integrin also plays a key role in arterial thrombosis, as demonstrated by the clinical use of antithrombotic drugs targeting αIIbβ3.^21^

Besides their key role in hemostasis and arterial thrombosis, platelets are also implicated in non-hemostatic functions such as embryonic and fetal development^22^^,^^23^ or immunity.^24^^,^^25^ There is solid experimental evidence that platelets contribute to tumor metastasis: tumor cells (TCs) colonized the lungs of mice much less efficiently in the context of severe thrombocytopenia.^26^ The importance of platelets in tumor growth and metastasis has been further confirmed in several experimental models.^27^^,^^28^^,^^29^^,^^30^^,^^31^ Platelets actively populate the tumor microenvironment where they modulate TCs proliferation, angiogenesis, and immune cell infiltration and function.^32^ Circulating platelets interact with TCs in the bloodstream, protecting them from shear forces^33^ and immune attacks,^27^ notably through inhibition of natural killer cells^34^ and the transfer of major histocompatibility complex class I molecules to the TCs.^35^ At distant sites, platelets favor adhesion of TCs to the endothelium^30^^,^^36^^,^^37^^,^^38^ and facilitate their extravasation,^30^^,^^39^^,^^40^ which favors metastatic colonization. Post-extravasation metastatic foci have been shown to be populated with platelets, where they further support metastatic growth through immune modulation.^30^ The action of platelets at distant sites is mediated by several receptors, including the GPIb-IX-V complex,^41^^,^^42^^,^^43^ CLEC-2,^44^^,^^45^^,^^46^^,^^47^^,^^48^ and GPVI.^30^^,^^49^^,^^50^

Platelet β1 and β3 integrins have been reported to contribute to tumor metastasis. We previously showed that mice lacking all platelet β1 integrins developed less lung metastasis in both an experimental metastasis model and an orthotopic model, with α6β1 playing a primary role.^31^ The involvement of this integrin could depend on its interaction with ADAM-9 expressed on TCs in the bloodstream,^31^ shielding them from shear stress and immune surveillance. Interestingly, the interaction of platelet α2β1 with MCF-7 breast cancer cells induces secretion of transforming growth factor β1, a cytokine known to promote epithelial-mesenchymal transition, suggesting that this integrin fuels in this way the invasive properties of TCs.^40^^,^^51^ In addition, αIIbβ3 has been reported to be implicated in tumor metastasis, as its inhibitors or deficiency decreased experimental metastasis.^52^^,^^53^^,^^54^^,^^55^^,^^56^ In fact, αIIbβ3 is able to interact with TCs through bridging with fibrinogen or vWF (interacting on the other side with αvβ3 expressed on the surface of TCs), thereby contributing to the formation of a shield around the TCs.

In this study, we characterize a mouse strain specifically deficient in all five platelet integrins (PF4Cre-β1^−/−^/β3^−/−^), which allows one to distinguish integrin-based adhesion from adhesion depending on other known receptors and to study the full contribution of platelet integrins by overcoming potential redundancies, known to be important for platelet integrins.^57^ After ensuring that depletion of integrins did not perturb the expression of other known platelet receptors, we assessed the combined role of integrins in platelet function, using flow cytometry and flow-based *in vitro* assays. Thus, we confirmed the essential contribution of integrins to hemostasis and thrombosis in bleeding time assays and arterial thrombosis models in mice. We could further demonstrate that these receptors actively participate in tumor growth and confirm their role in metastasis using orthotopic and experimental metastasis models.

## Results and discussion

### The central role of integrins in platelet function

**Figure 1:**
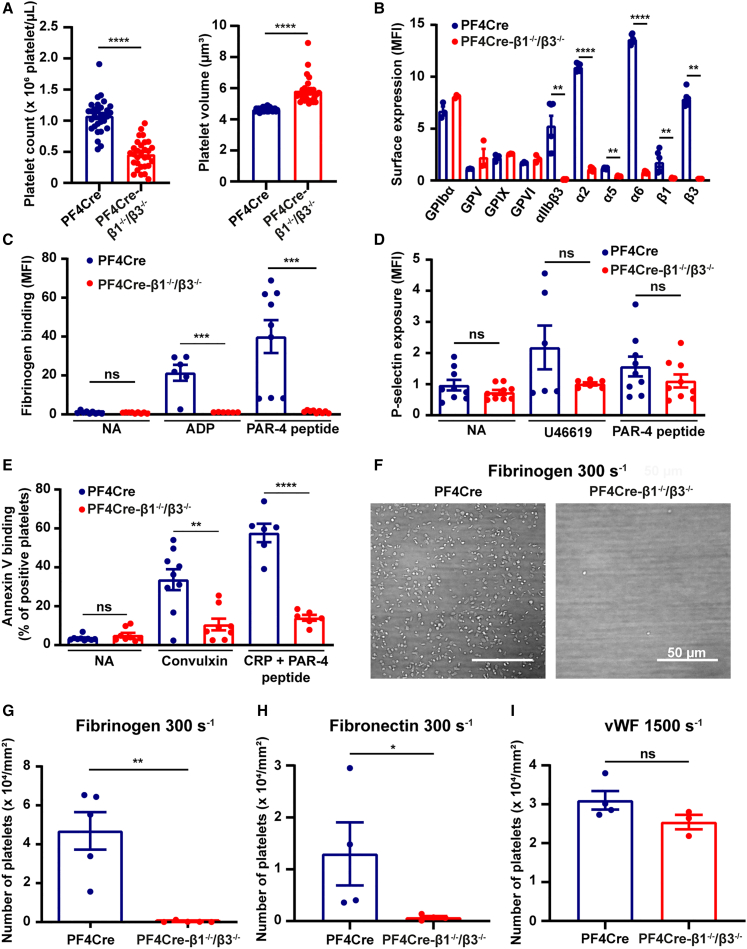
Platelet count, expression of major surface glycoproteins, and platelet function in PF4Cre-β1^−/−^/β3^−/−^ mice (A) Platelet count and volume. The platelet count and volume were determined in PF4Cre (*n* = 28–30) and PF4Cre-β1^−/−^/β3^−/−^ mice (*n* = 30). Data are from 6 independent experiments and were analyzed with an unpaired t test. (B) Glycoprotein surface expression. The surface expression of various glycoproteins on platelets in whole blood from PF4Cre (*n* = 3–6) and PF4Cre-β1^−/−^/β3^−/−^ mice (*n* = 3–6) was evaluated using selective antibodies and flow cytometry. Data are from 2 independent experiments and were analyzed with an unpaired t test or a Mann-Whitney test. (C) Fibrinogen binding. Whole blood from PF4Cre (*n* = 6–9) and PF4Cre-β1^−/−^/β3^−/−^ mice (*n* = 6–9) was stimulated for 10 min with ADP (2 μmol/L) or PAR-4 peptide (1 mmol/L) and the binding of fluorescein isothiocyanate (FITC)-fibrinogen was detected by flow cytometry. Data are from 3 independent experiments and were analyzed using a Mann-Whitney test. (D) P-selectin exposure. Whole blood from PF4Cre (*n* = 6–9) and PF4Cre-β1^−/−^/β3^−/−^ mice (*n* = 6–9) was stimulated for 10 min with U46619 (2 μmol/L) or PAR-4 peptide (1 mmol/L) and the binding of an FITC-conjugated anti-P-selectin antibody was detected by flow cytometry. Data are from 3 independent experiments and were analyzed with a Mann-Whitney test. (E) Annexin V binding. Whole blood from PF4Cre (*n* = 6–9) and PF4Cre-β1^−/−^/β3^−/−^ mice (*n* = 6–9) was stimulated with convulxin (15 nmol/L) or collagen-related peptide (CRP) (1 μg/mL) and PAR-4 peptide (1 mmol/L) for 10 min, incubated with Alexa Fluor 488-annexin V for 20 min and analyzed by flow cytometry. The forward light scattering and fluorescence intensity of 10,000 cells were collected with a logarithmic gain and the percentage of annexin V-positive platelets was determined in the upper quadrant of the plot. Data are from 6 independent experiments were analyzed using a Mann-Whitney test. (F–I) *In vitro* flow assays. Whole blood from PF4Cre (*n* = 4–5) and PF4Cre-β1^−/−^/β3^−/−^ mice (*n* = 3–5) was perfused through PDMS flow chambers coated with fibrinogen (100 μg/mL) at 300 s^−1^ for 5 min (F and G), through chambers coated with fibrillar cellular fibronectin (300 μg/mL) at 300 s^−1^ for 8 min (H), or through chambers coated with vWF A3 binding peptide vA3-III-23 (100 μg/mL) at 1,500 s^−1^ for 2 min (I). Platelet adhesion was visualized in random fields by DIC microscopy (F, scale bars, 50 μm), and the number of adherent platelets was quantified (G, H, and I). Data are from 2 or 3 independent experiments and were analyzed with an unpaired t test (I) or a Mann-Whitney test (G and H). Data information: results are expressed as the mean ± the standard error of the mean (SEM); ns *p* > 0.05; ∗*p* < 0.05; ∗∗*p* < 0.01; ∗∗∗*p* < 0.001; and ∗∗∗∗*p* < 0.0001. See also Figure S1.

To characterize the impact of the depletion of all platelet integrins, we took advantage of a mouse strain specifically deficient in platelet integrins (PF4Cre-β1^−/−^/β3^−/−^).^57^ PF4Cre-β1^−/−^/β3^−/−^ mice are viable and fertile but present reduced breeding, linked to an increased mortality during parturition. They have a 50% reduction in platelet count (PF4Cre: 10.7 × 10^5^ platelets/μL; PF4Cre-β1^−/−^/β3^−/−^: 4.58 × 10^5^ platelets/μL) and a 25% increase in mean platelet volume (PF4Cre: 4.64 μm^3^; PF4Cre-β1^−/−^/β3^−/−^: 5.82 μm^3^) as compared to control animals (Figure 1A). The surface expression of GPIbα, GPV, GPIX, and GPVI on platelets is normal while, as expected, the levels of all integrin subunits are profoundly reduced (Figure 1B). Using flow cytometry, we found that PF4Cre-β1^−/−^/β3^−/−^ platelets were unable to bind detectable levels of soluble fibrinogen in response to stimulation with adenosine diphosphate (ADP) or protease-activated receptor 4 peptide (PAR-4 peptide), due to the absence of αIIbβ3 (Figure 1C). In contrast, P-selectin exposure in response to the thromboxane A2 analog U46619 or PAR-4 peptide was completely normal, indicating no major role of platelet integrins in the secretion of α granule contents (Figure 1D). LAMP-1 expression was also not altered in the PF4Cre-β1^−/−^/β3^−/−^ mice as compared to controls, indicating normal lysosomal membrane fusion and exocytosis (Figure S1A). Additional experiments would be needed to evaluate the impact of platelet integrin deletion on dense granule secretion. Annexin V binding was markedly impaired in PF4Cre-β1^−/−^/β3^−/−^ mice as compared to controls (Figure 1E) but no reduction was observed in platelet-based thrombin generation (Figure S1B). This finding contradicts previous studies by other groups, which reported either no effect or an increase in procoagulant platelet formation in platelets from patients with Glanzmann’s thrombasthenia,^58^ as well as in platelets from healthy volunteers treated with αIIbβ3 antagonists.^59^ This difference could be the consequence of a combined deficiency in all platelet integrins implying a stronger phenotype. However, we also observed that tissue factor-mediated thrombin generation was normal with PF4Cre-β1^−/−^/β3^−/−^ blood, suggesting that it is unlikely that the defect in phosphatidylserine exposure translates into a functional effect *in vivo*. We further evaluated the ability of integrins to support platelet adhesion to different surfaces under flow conditions. Perfusing PF4Cre-β1^−/−^/β3^−/−^ mouse blood through microfluidic chips, we observed complete inhibition of platelet adhesion to fibrinogen or fibrillar fibronectin at wall shear rate of 300 s^−1^ (fibrinogen, PF4Cre: 4.69 ± 0.96 × 10^4^ platelets/mm^2^; PF4Cre-β1^−/−^/β3^−/−^: 0.03 ± 0.02 × 10^4^ platelets/mm^2^; fibrillar fibronectin, PF4Cre: 1.30 ± 0.61 × 10^4^ platelets/mm^2^; PF4Cre-β1^−/−^/β3^−/−^: 0.07 ± 0.03 × 10^4^ platelets/mm^2^) (Figures 1F–1H), indicating that β1 and β3 integrins are fundamental for platelets attachment and stable adhesion under relatively low arterial shear flows. In sharp contrast, platelet adhesion to immobilized plasma vWF at 1,500 s^−1^ was normal (PF4Cre: 3.10 ± 0.24 × 10^4^ platelets/mm^2^; PF4Cre-β1^−/−^/β3^−/−^: 2.54 ± 0.18 × 10^4^ platelets/mm^2^) (Figure 1I), confirming the modest contribution of integrins to the transient adhesion of platelets to vWF at high shear rates.^1^ Altogether, we could conclude that β1^−/−^/β3^−/−^ platelets were unable to bind to fibrinogen or fibrillar fibronectin under flow conditions.

### PF4Cre-β1^−/−^/β3^−/−^ mice exhibit a major defect in hemostasis and experimental thrombosis

**Figure 2:**
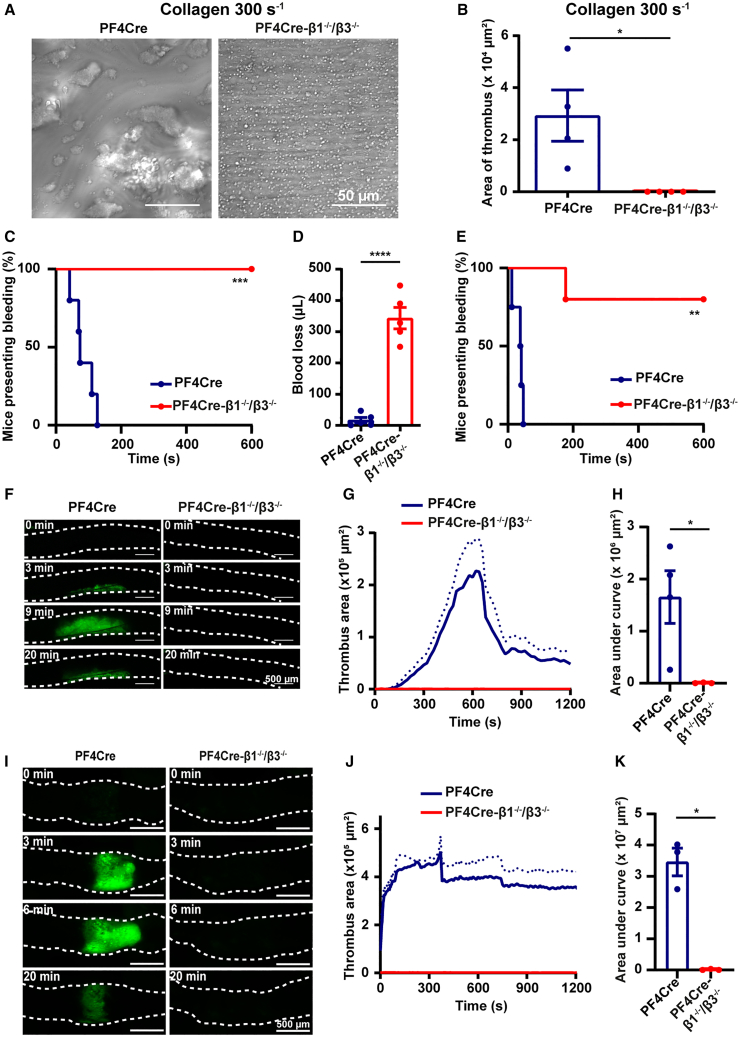
PF4Cre-β1^−/−^/β3^−/−^ mice are not able to form thrombosis and present an increased bleeding time (A and B) Thrombus formation *in vitro*. Whole blood from PF4Cre (*n* = 4) and PF4Cre-β1^−/−^/β3^−/−^ mice (*n* = 4) was perfused at 300 s^−1^ through PDMS flow chambers coated with collagen (200 μg/mL) for 8 min. Platelet aggregation was visualized in random fields by DIC microscopy (A, scale bars, 50 μm) and the area of the thrombi was quantified (B). Data are from 2 independent experiments and were analyzed using a Mann-Whitney test. (C and D) Tail bleeding time. The tail of PF4Cre (*n* = 5) and PF4Cre-β1^−/−^/β3^−/−^ mice (*n* = 5) was severed. The time required for the bleeding to stop was recorded (C) and the volume of blood lost was measured (D). Data are from 1 experiment and were analyzed with a Mantel-Cox test (C) or a Mann-Whitney test (D). (E) Carotid bleeding time. The carotid artery of PF4Cre (*n* = 4) and PF4Cre-β1^−/−^/β3^−/−^ mice (*n* = 4) was punctured and the time required for the bleeding to stop was recorded. Data are from 1 experiment and were analyzed with a Mantel-Cox test. (F–H) FeCl_3_-induced thrombosis of the carotid artery. Thrombosis was triggered in PF4Cre (*n* = 4) and PF4Cre-β1^−/−^/β3^−/−^ mice (*n* = 3) by placing a filter paper saturated with 7.5% FeCl_3_ on the common carotid artery. Representative fluorescence images of the thrombus (green) at the indicated time points after injury (F, scale bars, 500 μm). The areas of the thrombi were quantified (G) and the areas under the curves (AUC) were compared (H) using a Mann-Whitney test. Data are from 1 experiment. (I–K) Mechanically induced thrombosis of the abdominal aorta. Thrombosis was triggered in PF4Cre (*n* = 3) and PF4Cre-β1^−/−^/β3^−/−^ mice (*n* = 3) by pinching of the abdominal aorta with forceps. Representative fluorescence images of the thrombus (green) at the indicated time points after injury (I, scale bars, 500 μm). The areas of the thrombi were quantified (J) and the AUC were compared (K) using a Mann-Whitney test. Data are from 1 experiment. Data information: the symbols correspond to individual mice. Results are expressed as the mean ± the standard error of the mean (SEM); ∗*p* < 0.05; ∗∗*p* < 0.01; ∗∗∗*p* < 0.001; and ∗∗∗∗*p* < 0.0001.

To assess the combined role of platelet integrins in thrombus formation, anticoagulated whole blood from PF4Cre-β1^−/−^/β3^−/−^ mice was perfused at 300 s^−1^ through microfluidic chips coated with immobilized type-I collagen. Aggregation was abolished in PF4Cre-β1^−/−^/β3^−/−^ blood and only single platelet adhesion to the surface was observed (thrombus area, PF4Cre: 2.9 ± 1.0 × 10^4^ μm^2^; PF4Cre-β1^−/−^/β3^−/−^: 0.0 ± 0.0 × 10^4^ μm^2^) (Figures 2A and 2B). This result highlights the instrumental role of platelet integrins in thrombus formation under flow conditions, but also raises the question of how single platelets are able to stably adhere to collagen in the absence of α2β1, the best-known platelet adhesion receptor for collagen. We speculate that binding of the GPIb-IX-V complex to collagen-bound vWF, coupled to binding of GPVI to collagen, might explain the residual attachment of PF4Cre-β1^−/−^/β3^−/−^ platelets to the surface. Considering this major defect in thrombus formation, we then evaluated the bleeding time. Using a well-characterized mouse tail bleeding time model, we found that PF4Cre-β1^−/−^/β3^−/−^ mice presented a very marked increase in bleeding time, forcing us to stop the experiment at 600 s by cauterizing the injury to prevent the animals bleeding to death (Figure 2C). In addition, the volume of blood lost was much greater in PF4Cre-β1^−/−^/β3^−/−^ mice as compared to controls (PF4Cre: 17.0 ± 9.74 μL; PF4Cre-β1^−/−^/β3^−/−^: 344 ± 34.3 μL) (Figure 2D). These findings were confirmed in a second hemostasis model consisting of needle puncture of the carotid artery, which is much more severe than the classical tail bleeding assay (Figure 2E). The increase in bleeding time could not be explained by the 50% reduction in platelet count, as thrombocytopenic mice only exhibit prolonged bleeding when the platelet count is below 100,000 platelets/μL.^60^ These results confirmed the expected major role of platelet integrins in hemostasis in mice, especially that of αIIbβ3, consistent with the severe bleeding phenotype observed in Glanzmann thrombasthenia patients.^20^

Since we had only observed single platelet adhesion and no aggregation when perfusing PF4Cre-β1^−/−^/β3^−/−^ blood over collagen *in vitro*, we next evaluated the combined role of platelet integrins in two mouse models of arterial thrombosis: ferric chloride (FeCl_3_) injury of the carotid artery and mechanical injury of the abdominal aorta. In PF4Cre-β1^−/−^/β3^−/−^ mice, *in vivo* thrombus formation was profoundly inhibited with barely any sign of platelet adhesion at the site of lesion (area under the curve for the FeCl_3_ model, PF4Cre: 1.65 ± 0.51 × 10^6^ μm^2^; PF4Cre-β1^−/−^/β3^−/−^: 0.005 ± 0.005 × 10^6^ μm^2^; for mechanical injury: PF4Cre: 3.46 ± 0.44 × 10^7^ μm^2^; PF4Cre-β1^−/−^/β3^−/−^: 0.01 ± 0.01 × 10^7^ μm^2^) (Figures 2F–2K), highlighting the instrumental role of platelet integrins in thrombus formation *in vivo*. Similar to the bleeding time, these findings could not be explained by the 50% reduction in platelet count, as mice display normal experimental thrombosis with a platelet count above 300,000 platelets/μL in the model of mechanical lesion and above 200,000 platelets/μL in the model of FeCl_3_ injury.^60^ In summary, these results demonstrated that integrins are central to hemostasis and arterial thrombosis as they finely control platelet adhesion and aggregation at the site of vascular lesion.

### PF4Cre-β1^−/−^/β3^−/−^ mice present reduced primary tumor and metastatic growth

**Figure 3:**
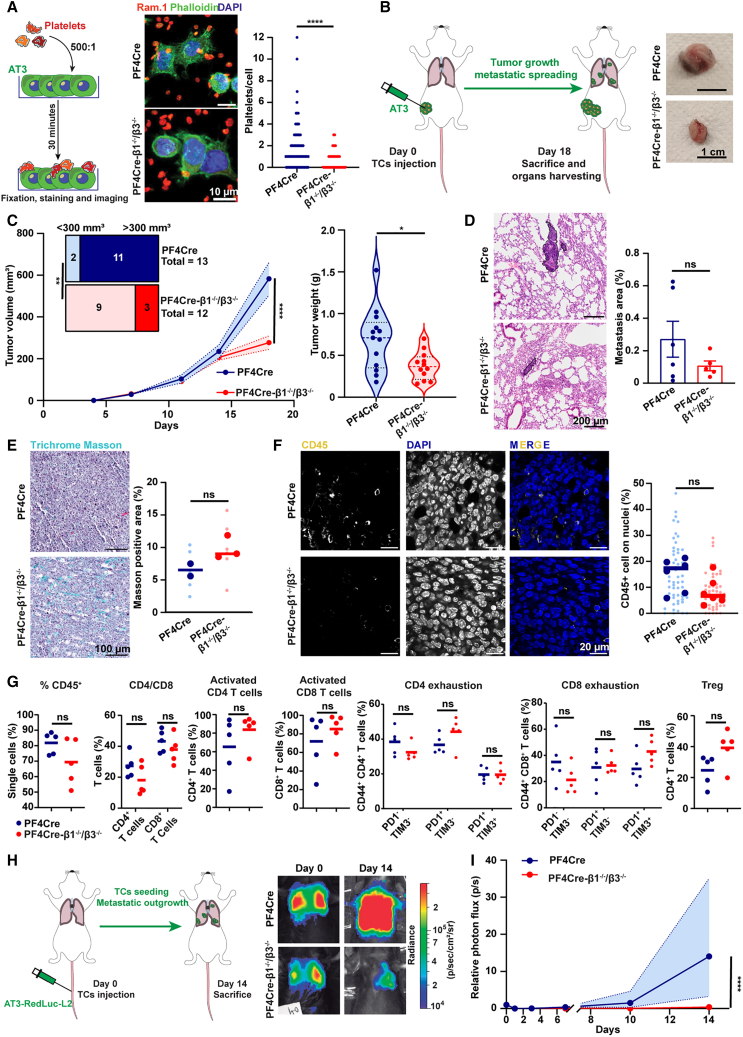
PF4Cre-β1^−/−^/β3^−/−^ platelets display a reduced binding to TCs and promote primary tumor growth and metastatic growth less efficiently (A) Platelet-tumor cell interactions *in vitro*. Left: infographics of the experiment. Middle: representative confocal microscopy images (scale bars, 10 μm). Right: graph representing the number of platelet interactions per tumor cell for PF4Cre (*n* = 356 cells) and PF4Cre-β1^−/−^/β3^−/−^ platelets (*n* = 288 cells). Data are from 1 experiment and were analyzed using a Mann-Whitney test after assessment of nonnormal distribution (Shapiro-Wilk test). (B) Orthotopic *in vivo* tumor model. Left: infographics of the experiment. Right: representative images of tumors in PF4Cre and PF4Cre-β1^−/−^/β3^−/−^ mice at sacrifice. Scale bars, 1 cm. (C) Orthotopic *in vivo* tumor model. Left: longitudinal measurement of the tumor *in vivo* (using Caliper) in PF4Cre (*n* = 13) and PF4Cre-β1^−/−^/β3^−/−^ mice (*n* = 12) during the experiment. The mean and range (dotted lines) are plotted. Data are from 2 independent experiments and were analyzed by two-way ANOVA. On the upper left, the numbers of mice with different tumor volumes were classified as indicated. Data are from 2 independent experiments and were analyzed by Fisher’s exact test. Right: *ex vivo* measurement of the weight of the tumor in PF4Cre (*n* = 13) and PF4Cre-β1^−/−^/β3^−/−^ mice (*n* = 12). The mean, quartiles, and data distribution are represented. Data are from 2 independent experiments and were analyzed with Welch’s test after assessment of normal distribution (Shapiro-Wilk test). (D) Hematoxylin and eosin staining on paraffin-embedded lungs from the orthotopic tumor model. Left: representative images (scale bars, 200 μm). Right: quantification of the percentage of metastatic area within the total tissue area in PF4Cre (24 images from 6 mice) and PF4Cre-β1^−/−^/β3^−/−^ mice (20 images from 5 mice). Each dot represents a single mouse and the mean ± SEM is reported for each experimental group. Data are from 1 experiment and were analyzed with Welch’s t test after assessment of normal distribution (Shapiro-Wilk test). (E) Masson Trichrome staining of tumors. Left: representative images (scale bars, 100 μm). Right: quantification of the percentage of Masson positive area in tumors from PF4Cre (6 images from 2 mice) and PF4Cre-β1^−/−^/β3^−/−^ mice (6 images from 3 mice). Each larger point represents the mean value of a single mouse; smaller points represent the mean of distinct images. The mean in each experimental group is reported. Data are from 1 experiment and were analyzed using a Mann-Whitney test after assessment of nonnormal distribution (Shapiro-Wilk test). (F) Immunofluorescence of CD45^+^ cells in paraffin-embedded tumors. Left: representative images (scale bars, 20 μm). Right: quantification of the percentage of CD45^+^ cells with respect to the number of nuclei in tumors from PF4Cre (55 images from 6 mice) and PF4Cre-β1^−/−^/β3^−/−^ mice (52 images from 6 mice). Each larger point represents the mean value in a single mouse; smaller points represent the mean of distinct images. The mean in each experimental group is reported. Data are from 1 experiment and were analyzed with an unpaired t test after assessment of normal distribution (Shapiro-Wilk test). (G) *Ex vivo* immunophenotyping of tumors by flow cytometry. The percentages of the immune cell populations within the defined gates are represented for tumors from PF4Cre (*n* = 5) and PF4Cre-β1^−/−^/β3^−/−^ mice (*n* = 6). Data are from 1 experiment and were analyzed using an unpaired t test or a Mann-Whitney test according to distribution normality (Shapiro-Wilk test). (H) *In vivo* experimental metastasis model. Left: infographics of the experimental. Right: representative images of the lung body luminescence signal (BLI) on 0 and 14 days post injection (dpi). (I) Body luminescence index (BLI) values from the experimental metastasis model. The relative photon flux (p/s) as compared to day 0 is represented for PF4Cre (*n* = 8) and PF4Cre-β1^−/−^/β3^−/−^ mice (*n* = 6). The mean and range are plotted. Data are from 1 experiment and were analyzed by two-way ANOVA. Data information: Results are expressed as dot plot, the bar indicated the mean; ns *p* > 0.05; ∗*p* < 0.05; ∗∗∗∗*p* < 0.0001. See also Figure S2.

We next looked at whether combined integrin deficiency could alter platelet-dependent tumor phenotypes. Interestingly, adhesion of integrin-deficient platelets to triple-negative breast cancer cells (AT3 cells) was strongly reduced (PF4Cre-β1^−/−^/β3^−/−^: 0–3 platelets/cell; PF4Cre: 0–12 platelets/cell) (Figure 3A) prompting us to investigate whether this could affect tumor progression. Platelets are a component of the tumor microenvironment and support primary tumor growth.^32^^,^^61^^,^^62^ We first assessed the impact of combined platelet β1 and β3 integrins deficiency on primary tumor growth using a syngeneic orthotopic model (AT3 cells) in PF4Cre-β1^−/−^/β3^−/−^ and PF4Cre mice (Figure 3B). Mammary tumor volume and weight were significantly decreased in PF4Cre-β1^−/−^/β3^−/−^ as compared to control animals (Figure 3C). In addition, PF4Cre mice present a significant higher number of tumor with a volume >300 mm^3^ (11/13 mice) as compared to PF4Cre-β1^−/−^/β3^−/−^ mice (3/12 mice) (Figure 3C). Lung metastasis was similarly perturbed in PF4Cre-β1^−/−^/β3^−/−^ mice showing a 60% reduction compared to PF4Cre mice, although not reaching statistical significance (Figure 3D). This suggests that platelet integrin deficiency also impairs metastatic spreading of TCs, likely through a combined effect due to reduced platelet-TC binding (Figure 3A), diminished tumor growth (Figures 3B and 3C) and 50% reduction of platelet count (Figure 1A). Tumors in mice lacking platelet integrins displayed a tendency toward increased collagen deposition (Figure 3E). This suggested that the absence of integrins on platelets alters their ability to organize the tumor-associated stroma, as seen in light of the known role of platelets in ECM deposition^63^ and integrin αIIbβ3 recognition as a driver of fibronectin fibrillogenesis *in vitro.*^64^

In addition to these structural components, the tumor microenvironment also comprises tumor-associated cells including immune cells. Considering the well-established feedforward communication between platelets and the immune microenvironment of the tumor,^32^ we further investigated this compartment by tissue immunofluorescence and observed a trend toward reduced infiltration of CD45^+^ cells in PF4Cre-β1^−/−^/β3^−/−^ tumors (Figure 3F). Additional analyses using *ex vivo* multiparametric flow cytometry (gating strategy presented in Figure S2A) confirmed that PF4Cre-β1^−/−^/β3^−/−^ tumors exhibited a trend toward a lower content of CD45^+^ cells (Figure 3G). Within the myeloid panel, only neutrophils displayed accumulation in PF4Cre-β1^−/−^/β3^−/−^ mice (Figure S2B). In the lymphoid lineage, while there was no variance in total T cells, relative numbers of CD4^+^ and CD8^+^ cells were slightly reduced in PF4Cre-β1^−/−^/β3^−/−^ tumors (Figure 3G). Whereas a decrease in the PD1^−^ TIM3^−^ compartment was observed in both CD4^+^ and CD8^+^ cells (Figure 3G), PD1^+^ TIM3^−^ interferon-producing effector/exhausted cells^65^^,^^66^ were exclusively increased in the CD4^+^ subgroup (Figure 3G). Moreover, memory CD8^+^ T cells from PF4Cre-β1^−/−^/β3^−/−^ mice presented a significant increase in PD-1 expression (MFI) (Figure S2C), which, in this T cell subset, is required to maintain the memory T cell pool.^67^ In addition, TIM-3 expression was also augmented in terminally exhausted effector PD-1^+^ CD8^+^ T cells from PF4Cre-β1^−/−^/β3^−/−^ compared to control mice (Figure S2C). Lastly, T regulatory cells were increased in PF4Cre-β1^−/−^/β3^−/−^ mice (Figure 3G). While these data further consolidate the role of platelets in immune cells recruitment at tumor sites,^68^ they demonstrate a non-negligible contribution of platelet integrins, as suggested by previous studies showing that platelets could promote anti-tumor T cell functions through microparticle adoptive transfer of β3 integrin in a hepatocellular carcinoma model.^69^ Alternatively, platelet integrins might alter the surface expression of immune checkpoint molecules, thereby remodeling the T cell response, and ITGB2A expression has been correlated, among the others, to PDL1, CD80, and CD86 expression in humans (METABRIC cohort).^68^ Overall, our data point to a critical involvement of combined platelet β1 and β3 integrin deficiency in impairing primary tumor growth, ECM deposition and immune cells recruitment and function.

Although the mechanistic control of the recruitment of immune components by platelets requires additional investigation,^68^ the latter are known to bind to circulating TCs in the bloodstream, where they promote metastasis.^30^^,^^70^ Since the orthotopically implanted tumors of PF4Cre-β1^−/−^/β3^−/−^ mice displayed a trend toward reduced metastatic potential (Figure 3D), under conditions where binding of β1^−/−^/β3^−/−^ platelets to TCs was significantly reduced (Figure 3A), we were able to set up an experimental metastasis model aimed at directly testing the contribution of platelet integrins to the hematogenous phase of metastasis (Figure 3H). As expected, the metastatic growth of AT3 cells was significantly impaired in PF4Cre-β1^−/−^/β3^−/−^ mice at 14 days post-injection (dpi) (Figure 3I). This could be the consequence of the absence of β1 and β3 integrins on platelets, but might also be linked to the reduction of the platelet count observed in PF4Cre-β1^−/−^/β3^−/−^ mice. We recently showed that reducing platelet counts had a significant impact on metastasis in two syngeneic models of experimental lung metastasis^30^ and that a 70% reduction of the platelet count leads to a 50% reduction in lung metastasis formation in the experimental metastasis model.^31^ However, another publication observed that a 60% reduction of the platelet count does not have a significant impact on lung metastasis development,^29^ suggesting that the reduction in lung metastasis could indeed be a direct consequence of the absence of platelet integrins. While this result recapitulates previously observations made in platelet β1 deficiency^31^ and β3 deficiency^52^^,^^54^^,^^55^^,^^56^ or pharmacological blockade of platelet integrins,^52^^,^^53^^,^^54^^,^^55^^,^^56^ it emphasizes the central role of platelet β1 and β3 integrins in mediating platelet-TC interactions and subsequent metastatic progression.

In conclusion, this study characterizes PF4Cre-β1^−/−^/β3^−/−^ mice and assesses the effect of depletion of all platelet integrins on hemostasis, thrombosis, and tumor growth. While the surface expression of integrins was abolished, PF4Cre-β1^−/−^/β3^−/−^ platelets presented normal glycoprotein expression and P-selectin exposure, indicating no major role of platelet integrins in α granule content secretion. Conversely, we provide evidence for a key role of integrins in many platelet functions including procoagulant activity and platelet attachment and stable adhesion at relatively low shear rates. In addition, aggregation on collagen under flow conditions was completely inhibited in the absence of platelet integrins, a result that was confirmed *in vivo* by the total prevention of thrombus formation in two distinct models of arterial thrombosis. PF4Cre-β1^−/−^/β3^−/−^ mice displayed severely increased bleeding in two different models of the bleeding time, probably due to the absence of αIIbβ3, which reflects the severe hemorrhagic phenotype observed in Glanzmann’s thrombasthenia patients. Moreover, we show that platelet integrins participate in platelet-TC interactions. Integrin deficiency reduced primary tumor growth and to some extent pulmonary metastatic dissemination in a syngeneic AT3 model, affecting ECM deposition and immune cells accumulation and functionality at the tumor site. PF4Cre-β1^−/−^/β3^−/−^ mice also presented diminished metastatic growth in an experimental metastasis model. Altogether, our study sheds light on the irreplaceable functions of platelet integrins in controlling hemostasis and arterial thrombosis, and we demonstrate their non-negligible participation in shaping primary and metastatic tumor growth.

### Limitations of the study

This study confirms the central role of platelet integrins in hemostasis and thrombosis, and highlights their contribution to tumor growth and metastasis formation. However, the role of each of the five platelet integrins in tumor growth and metastasis was not addressed. Further studies should explore the role of individual integrins in these processes to provide more comprehensive understanding of their implication. While we demonstrated an important involvement of these integrins in diminishing primary tumor growth, their mechanistic control of the immune components’ recruitment there and in tumor metastasis requires additional investigation.

## Resource availability

### Lead contact

Further information and requests for resources and reagents should be directed to and will be fulfilled by the lead contact, Emily Janus-Bell (emily.janus-bell@efs.sante.fr).

### Materials availability

This study did not generate new unique reagents or cell lines.

### Data and code availability

• All data reported in this paper will be shared by the lead contact upon reasonable request.
• This article does not report original code.
• Any additional information about the data reported in this paper will be shared by the lead contact upon request.

## Acknowledgments

This work was supported by INSERM, EFS, and ARMESA. We would like to acknowledge the PICSTRA imaging platform for their technical assistance during imaging acquisition, Marina Peralta for the Fiji analysis pipeline design, and the CytoTriCS platform for their technical assistance during flow cytometry experiments. Our work was also supported by grants from the French National Cancer Institute to P.H.M. and J.G.G. (PLBIO2023-173), which covered the financial support for C.L. V.M. was the recipient of a fellowship from the French Ministry of Science (MESRI) and a fourth-year thesis fellowship from the Foundation ARC-Association pour la Recherche sur le Cancer. L.B. was the recipient of a PhD scholarship from the FRM-Fondation pour la Recherche Médicale. Parts of the figures were drawn using material from Servier Medical Art, which is licensed under a Creative Commons Attribution 3.0 Unported License (https://creativecommons.org/licenses/by/4.0/).

## Author contributions

E.J.-B. and C.L. were in charge of investigation, formal analysis, validation, visualization, and writing the paper (original draft, reviewing and editing). A.Y., V.M., and O.L. contributed to investigation, formal analysis, visualization, and writing the paper (original draft). C.M., C.B., L.B., V.H., and M.G.-L. contributed to investigation and formal analysis. P.H.M. and J.G.G. were in charge of conceptualization, funding acquisition, project administration, supervision, and writing the paper (original draft, reviewing and editing).

## Declaration of interests

The authors declare no competing interests.

## STAR★Methods

### Key resources table

**Table:** 

REAGENT or RESOURCE	SOURCE	IDENTIFIER
**Antibodies**		

FITC-conjugated anti-αIIbβ3 (clone Leo.F2)	Emfret Analytics	Cat. M025-1; RRID:AB_2833085
FITC-conjugated anti-GPIbα (clone Xia.G7)	Emfret Analytics	Cat. M042-1; RRID:AB_2827527
FITC-conjugated anti-GPV (clone Gon.G6)	Emfret Analytics	Cat. M061-1
FITC-conjugated anti-GPIX (clone Xia.B4)	Emfret Analytics	Cat. M051-1; RRID:AB_2827529
Anti-GPVI (clone Jaq.1)	Emfret Analytics	Cat. M011-1; RRID:AB_2827531
FITC-conjugated anti-β3 (clone Luc.H11)	Emfret Analytics	Cat. M031-1; RRID:AB_2827528
PE-conjugated anti-α2 (clone HMα2)	BioLegend	Cat. 103506; RRID:AB_313029
PE-conjugated anti-α5 (clone 5H10-27)	BioLegend	Cat. 103805; RRID:AB_313054
PE-conjugated anti-α6 (clone GOH3)	BioLegend	Cat. 313612; RRID:AB_893373
Alexa Fluor 488-conjugated anti-β1 (clone HMβ1-1)	BioLegend	Cat. 102211; RRID:AB_492830
FITC-coupled anti-P-selectin (clone RB40.34)	BD Pharmingen	Cat. 561923
PE/Dazzle594 anti-CD103 (clone 2E7)	BioLegend	Cat.121430; RRID:AB_2566493
PerCP/Cy5 anti-CD11b (clone M1/70)	BioLegend	Cat. 101211; RRID:AB_312794
PE anti-CD11c (clone N418)	BioLegend	Cat. 117308; RRID:AB_313777
APC anti-CD138 (clone, 281-2)	BioLegend	Cat. 558626
BV711 anit-CD19 (clone 6D5)	BioLegend	Cat. 115554; RRID:AB_2564001
BV421 anti-CD25 (clone A18246A)	BioLegend	Cat. 113705; RRID:AB_3068095
PerCP/Cy5 anti-CD3 (clone 17A2)	BioLegend	Cat.100218; RRID:AB_1595492
Alexa Fluor 700 anti-CD4 (clone RM4-5)	BioLegend	Cat. 100536; RRID:AB_493701
FITC anti CD62L (clone MEL-14)	BD Pharmingen	Cat. 553150
BV510 anti-CD45 (clone 30-F11)	BioLegend	Cat.103138; RRID:AB_2563061
BV605 anti-CD8 (clone 53–6.7)	BioLegend	Cat.100744; RRID:AB_2562609
PE anti-CTLA4/CD152 (clone UC10-4B9)	BioLegend	Cat. 106306; RRID:AB_313255
PE/Cy7 anti-F4/80 (clone BM8)	BioLegend	Cat.123114; RRID:AB_893478
PE/Cy7 anti-IgD (clone 11-26c.2a)	BioLegend	Cat. 405720; RRID:AB_2561876
Alexa Fluor 488 anti-Ly6C (clone HK1.4)	BioLegend	Cat. 128022; RRID:AB_10639728
BV711 anti-Ly6G (clone 1A8)	BioLegend	Cat. 127643; RRID:AB_2565971
Alexa Fluor 700 anti-MHCII (clone M5/114.15.2)	BioLegend	Cat. 107622; RRID:AB_493727
BV421 anti-NK1.1 (clone PK136)	BioLegend	Cat. 108731; RRID:AB_10895916
PE/Dazzle594 anti-PD-1/CD279 (clone 29F.1A12)	BioLegend	Cat. 135228; RRID:AB_2566006
TruStain FcX™ (anti CD32/16 antibody)	BioLegend	Cat. 101319; RRID:AB_1574973
PE anti-CD38 (clone 90/CD38)	BD Pharmingen	Cat. 553764
PE anti-CD44 (clone IM7)	BD Pharmingen	Cat. 553134
PE/Dazzle594 anti-CD80 (clone 16-10A1)	BD Pharmingen	Cat. 562504
Alexa Fluor 647 anti-TCF-1 (clone S33-966)	BD Pharmingen	Cat. 566693
FITC anti-FoxP3 (clone FJK-16S)	eBioscience	Cat. 53-5773-82
PE/Cy7 anti-TIM-3/CD366 (clone RMT3-23)	eBioscience	Cat. 25-5870-82
Anti-mouse CD45 biotinylated (clone#30-F11)	BioLegend	Cat.103103; RRID:AB_312968
Streptavidin-AF647	BioLegend	Cat. 405237
Alexa Fluor 647 coupled anti-RAM.1	EFS Strasbourg	N/A

**Chemicals, peptides, and recombinant proteins**		

U46619	Sigma-Aldrich	Ref. D8174
PAR-4 peptide	Sigma-Aldrich	Ref. TA9H93ED6C65
Polydimethylsiloxane (PDMS) Sylgrad 184 Silicone elastomer base and curing agent	Sigma-Aldrich	Ref. 01673921
Cellular fibronectin	Sigma-Aldrich	Ref F2518
Alexa Fluor 488-coupled fibrinogen	Molecular Probes	Cat. F-13191
DIOC_6_ (3,3′-dihexyloxacarbocyanine iodide)	Invitrogen	Ref D273
Ethylenediaminetetraacetic acid (EDTA)	Invitrogen	Ref. 15575-038
iFluor 488-Phalloidin	Sigma	Ref P1951-1 MG
APC-Cy7-Viability Zombie NIR	BioLegend	Ref. 423106
Annexin V-Alexa Fluor488	Life technologies	Ref A13201
eBioscience™ FOXP3/Transcription factor staining buffer set	Invitrogen	Cat. 00-5523-00
Recombinant hirudin	Transgene	N/A
Alexa Fluor 488-annexin V	Life Technologies	Cat. A13201
Paraformaldehyde (PFA)	Electron microscopy sciences	Ref. 15714
Eukitt mounting medium	Electron microscopy sciences	Cat.15320
ADP	Mast group	N/A
Convulxin	Cryoprep	Ref. 8-119-02
Fibrinogen	Intertransfusion	N/A
vWF A3 binding peptide	Cambcol	vA3-III-23
Collagen (Kollagenreagens Horm)	Takeda	Ref. 1130630
Ferric chloride (FeCl_3_)	Prolabo	Ref. 24 212.298
Luciferin	Perkin-Elmer	Ref. 760504
Harris Hematoxylin Acidified	Epredia	Ref. 6765003
Eosin Y alcoholic	Epredia	Ref. 6766007
Fluoromount-G™ with DAPI	Fischer Scientific	Cat. 00-4959-52

**Critical commercial assays**		

Avidin/biotin blocking kit	Vector Laboratories	Cat. SP-2001
Masson Trichrome kit, light green variation	CellaVision-RalDiagnostics	Cat. 361350-0000
Tumor Dissociation kit-mouse	Miltenyi	Cat. 130-096-730

Deposited data		

Depletion of all platelet integrins impacts hemostasis, thrombosis and tumor metastasis	BioRxiv	https://doi.org/10.1101/2024.11.22.624871

**Experimental models: Cell lines**		

Mouse: AT3-RedLuc-L2 (AT3) cells	Garcia-Leon et al.^30^	N/A

**Experimental models: Organisms/strains**		

Mouse: PF4Cre-β1^−/−^/β3^−/−^ (C57BL/6J background)	UMRS1255	N/A
Mouse: PF4-Cre^+^ (called PF4Cre) (C57BL/6J background)	Radek C. Skoda	N/A

**Software and algorithms**		

Prism	GraphPad Prism software 9.5.0	
ImageJ	Fiji (Schindelin et al.^71^)	
Metamorph	Molecular Devices	
QuPath 0.5.0	Bankhead et al.^72^	
FlowJo™ v10 Software	ThreeStar	
Living Image® software	Perkin-Elmer	Version 4.7.3

### Experimental model and study participant details

#### Cell line

A murine triple negative breast cancer cell line (AT3) was previously established in our laboratory.^30^ The cells were cultured at 37°C under 5% CO_2_ in RPMI 1640 medium supplemented with 10% FBS and 1% penicillin/streptomycin under puromycin selection (1 μg/mL). At confluency, the cells were trypsinized and sub-cultured for further passages. The AT3 cell line was authenticated based on its tumor growth on a C57BL/6J mouse genetic background. This cell line was regularly tested for the absence of mycoplasma contamination.

#### Animal experiments

Adult C57BL/6J mice lacking platelet β1 and β3 integrins (called PF4Cre-β1^−/−^/β3^−/−^)^57^ were used with adult PF4-Cre^+^ (called PF4Cre) animals serving as controls. Food and water were provided *ad libitum* and the light/dark cycle duration was 10h of light. Sex and gender do not have an influence on the results allowing male and female mice to be employed except for tumor models with a murine triple negative breast cancer cell line where only females were used because of the nature of the TCs. All procedures for animal experiments were approved by the regional ethical committee for animal experimentation and the French government (animal facility authorization: G67-482-10 and APAFIS 27659–2020101308518816, C67-482-33 and APAFIS 37433–2022052016445806).

### Method details

#### Platelet count and volume

After severing the tail of anesthetized mice, whole blood was collected into EDTA (6 mM). The platelet count and volume were analyzed in an automatic cell counter (Element HT5, Heska).

#### Platelet glycoprotein expression

After severing the tail of anesthetized mice, whole blood was collected into EDTA (6 mM). The blood was diluted in PBS to obtain 100,000 platelets/μL and incubated with labeled murine antibodies for 30 min at room temperature (anti-αIIbβ3 1:20; anti-GPIbα 1:20; anti-GPV 1:20; anti-GPIX 1:20; anti-GPVI 1:100; anti-β3 1:20; anti-α2 1 μg/mL; anti-α5 2 μg/mL; anti-α6 1 μg/mL; anti-β1 1 μg/mL). The samples were then diluted in 500 μL of PBS and surface glycoprotein expression (10,000 platelet events) was determined by flow cytometry (Gallios, Beckman Coulter).

#### Platelet fibrinogen and annexin V binding, P-selectin exposure

Whole blood was drawn into hirudin anticoagulant (100 U/mL final concentration) from the abdominal aorta of anesthetized mice. For soluble fibrinogen binding and P-selectin exposure, the blood was diluted in Tyrode albumin (0.35%) buffer containing hirudin (100 U/mL), incubated with Alexa Fluor 488-conjugated fibrinogen (20 μg/mL) or an FITC-coupled anti-P-selectin antibody (2.5 μg/mL) and stimulated with agonists (ADP 2 μmol/L, U46619 2 μmol/L or PAR-4 peptide 1 mmol/L) for 10 min. Samples were fixed by adding 50 μL of 4% PFA for 20 min and then diluted in 600 μL of PBS. For phosphatidylserine exposure, the blood was diluted in tyrode albumin (0.35%) buffer containing hirudin (100 U/mL), stimulated with agonists (Convulxin 15 nmol/L or collagen-related peptide 1 μg/mL and PAR-4 peptide 1 mmol/L) for 10 min and then incubated with Alexa Fluor 488-annexin V (1 μg/mL) for 20 min in the presence of hirudin. Fluorescence (10,000 platelet events) was determined by flow cytometry (Gallios, Beckman Coulter).

#### *In vitro* flow based assay

Flow experiments were performed as previously described.^16^ PDMS flow chambers (0.1 × 1 mm) were coated with fibrinogen (100 μg/mL), cellular fibronectin (300 μg/mL), vWF A3 binding peptide vA3-III-23 (100 μg/mL) or collagen (200 μg/mL) overnight at 4°C. To ensure mechanical stretching of cellular fibronectin, two silicone tubes were connected to vacuum pumps providing a vacuum of 100 mbar. The tubes were clamped and after 1 min, connected to the inlet and outlet of the flow chamber. The clamps were then opened to apply a tensile force to the surface and allow the fibronectin to multimerize through mechanical stretching. The coated flow channels were blocked with PBS containing HSA (10 mg/mL) for 30 min at room temperature to limit nonspecific platelet adhesion. Hirudinated (100 U/mL) whole blood drawn from the abdominal aorta of anesthetized mice was perfused through the channels with a programmable syringe pump (Harvard Apparatus, PHD 2000) at 300 s^−1^ (over fibrinogen, fibronectin and collagen) or at 1,500 s^−1^ (over vWF A3 binding peptide). Platelet adhesion and thrombus formation were monitored by DIC microscopy (Leica DMI4000B) using a 63× or 40× objective and a Hamamatsu ORCA Flash 4L.T camera (Hamamatsu Photonics). The number of platelets adhering or the area of the thrombus was quantified using Fiji software.^71^

#### Bleeding time

The tail bleeding time was determined by severing 3 mm from the tip of the tail of anesthetized mice. The tails were immersed in 0.9% saline at 37°C and the time required for the bleeding to stop was recorded. After centrifuging the tubes containing the blood for 5 min, the pellets were homogenized in lysis buffer (NH_4_Cl 150 mM, KHCO_3_ 1 mM, EDTA 0.1 mM, pH 7.2) and used for quantification of the blood lost by comparing the optical density at 540 nm with a standard curve. Standard values were established using known quantities of blood in 50 μL, diluted in 450 μL of lysis buffer. The bleeding time was also measured by puncture of the carotid artery of anesthetized mice with a 25 G needle. The time to cessation of bleeding was determined using a fluorescent macroscope (Leica Microsystems) and a charge-coupled device (CCD) camera (ORCA-Fusion C14440-20UP, Hamamatsu). Image acquisition was performed with Metamorph software (Molecular Devices) and data analysis with Fiji.^71^

#### *In vivo* thrombosis models

Mice were anesthetized and received an injection of DIOC_6_ to label platelets. For the FeCl_3_ model, after exposing the left common carotid artery, a vascular injury was induced by applying a 3 × 3 mm Whatman filter paper saturated with 7.5% FeCl_3_ laterally to the carotid for 2.5 min. For the mechanical model, a lesion was induced by pinching the abdominal aorta with forceps for 15 s. Thrombus formation was monitored for 20 min with a fluorescent macroscope (Leica Microsystems) coupled to a CCD camera (ORCA-Fusion C14440-20UP, Hamamatsu). Image acquisition was performed with Metamorph software (Molecular Devices) and analysis of the thrombus size with Fiji.^71^

#### Platelet-tumor cell interactions

To study platelet-TC interactions, AT3-RedLuc-L2 (AT3) cells were seeded (6,000 per well) in a 12-well chamber and left to adhere overnight in complete medium. The following day, platelets (citrated platelet-rich plasma prepared as described by Garcia-Leon et al.^30^) were added to each well at a concentration of 500 platelets per cell and let to interact for 30 min at 37°C under 5% CO_2_ in Tyrode’s buffer without MgCl_2_. After three washes in PBS, the cells were fixed in 0.5% PFA in PBS for 20 min at room temperature. After three further washes in PBS, the cells were stained with Alexa Fluor 647-coupled anti-RAM.1 (2 μg/mL) and iFluor 488-Phalloidin (1:1000) for 1 h at room temperature. The slides were then washed and mounted in Fluoromount-G with DAPI. Images were acquired with a 60×/NA1.2 water-objective using an Olympus Spinning disk confocal microscope. Data analysis was performed with Fiji software.^71^ The number of cells interacting with platelets was manually counted.

#### Syngeneic orthotopic tumor model

AT3 syngeneic triple negative breast cancer cells (250,000) were intraductally injected into PF4Cre-β1^−/−^/β3^−/−^ and control (PF4Cre) mice. The tumor dimensions were measured longitudinally for up to 18 days post-injection (dpi) using Caliper and the tumor volume was calculated with the following formula: volume = width^2^ x length. At sacrifice of the mice, the tumors were excised, weighted and processed for *ex vivo* flow cytometric immunophenotyping or immunofluorescence/Masson staining. At the same time, the lungs were harvested and processed for hematoxylin/eosin staining.

#### Syngeneic experimental metastasis model

AT3 syngeneic triple negative breast cancer cells (150,000) were injected into the lateral vein of the tail of PF4Cre-β1^−/−^/β3^−/−^ and control (PF4Cre) mice. Metastatic growth was followed longitudinally *in vivo* by intraperitoneal injection of luciferin (5 μg/kg) and imaging with IVIS Lumina III (PerkinElmer).^30^ The animals were sacrificed 14 dpi. IVIS results were analyzed with Living Image software, plotting the ratio of the photon emission as compared to day 0, as previously described.^30^

#### Processing of mouse organs

Tumors and lungs were harvested and fixed in 4% PFA in PBS overnight and processed for paraffin inclusion as previously described.^30^ Briefly, after two successive washes in PBS and 50% ethanol, the samples were dehydrated overnight in 70% ethanol. The tissues were then embedded in paraffin in an automated Leica inclusion machine according to the following schema: ethanol baths (2× at 70%, 1× at 80%, 1× at 95% and 2× at 100%) 1 h each, 2 xylene baths (1 h each) and 2 paraffin embedding baths (1 h each).

#### Hematoxylin and eosin staining

Lung sections (10 μm) were dewaxed and rehydrated in decreasing alcohol solutions until 70% ethanol. The slides were then rinsed under a continuous stream of tap water for 5 min. Hematoxylin staining was carried out in Harris hematoxylin acidified for 3 min and followed by washing under a continuous stream of tap water for 5 min. The staining was differentiated with a quick wash in acid alcohol (1% HCl +70% ethanol) and rinsing again under a continuous stream of water for 5 min. Counterstaining was carried out in eosin for 15 s and followed by washing again under a continuous stream of water for 5 min. The sections were then dehydrated in increasing alcohol solutions until a final bath in xylene and mounted in Eukitt mounting medium. Images were acquired with a Slide Scanner VS200 microscope (Olympus) using a 20×/NA0.8 air-objective. Analyses were performed with QuPath 0.5.0 software.^72^ Metastastic areas were manually annotated as hematoxylin-dense areas and the percentage of metastatic areas was calculated over the total tissue area (automatically annotated via eosin signal thresholding).

#### Immunofluorescence staining and analysis

Sections (10 μm) were cut from paraffin-embedded tumors with a Leica microtome and mounted on Superfrost slides. The tissues were dewaxed and rehydrated in decreasing alcohol solutions and antigens were demasked by boiling in Citrate buffer pH 6.0 Antigen Retriever.^30^ After background minimization in blocking solution (3% BSA, 20 mM MgCl_2_, 0.3% Tween 20, 5% FBS in PBS) and endogenous avidin/biotin blockade, the slides were incubated with the primary anti-mouse CD45 biotinylated antibody (1:100) overnight at 4°C. The following day, they were incubated with Streptavidin-AF647 (1:200, Sigma) diluted in 5% BSA for 1 h at room temperature. The samples were then mounted in Fluoromount-G with DAPI. Images were acquired with a 40×/oil objective using a Zeiss LSM800 confocal microscope and analyses were performed with Fiji software.^71^ The number of nuclei was automatically counted using the “analyze particles” plug-in after subtraction of the background and thresholding of the images. The number of CD45^+^ cells was manually counted on the processed images and the percentage of CD45^+^ cells with respect to the number of nuclei was expressed.

#### Masson Trichrome staining and analysis

Paraffin-embedded tumors were cut into 5 μm sections with a Leica microtome and mounted on Superfrost slides. After dewaxing and rehydration in a decreasing alcohol scale, the tissues were stained with a Masson Trichrome kit, light green variation, according to the manufacturer’s instructions modified by using 5 min mordant and 10 min staining incubation times. The samples were then dehydrated in increasing alcohol solutions with two final baths in xylene and mounted in Eukitt medium. Images were acquired with a Slide Scanner VS200 microscope (Olympus) using a 20×/NA0.8 air-objective. Analyses were performed with QuPath 0.5.0 software.^72^ The tissue area was thresholded and automatically annotated in the blue channel at high resolution (1.10 μm/px), while the Masson positive area was thresholded and automatically annotated in the residual channel at very high resolution (0.55 μm/px). The analysis was manually corrected to remove tissue artifacts. The percentage of Masson positive area was calculated with respect to the total tissue area.

#### Tumor immunophenotyping using *ex-vivo* flow cytometry

A single cell suspension was obtained from freshly harvested tumors as described by Garcia Leon et al*.*^30^ Briefly, 2 mm^3^ pieces of tumor were cut before being enzymatically digested using a Tumor Dissociation kit mouse diluted in RPMI 1640 according to the manufacturer’s instructions. Digestion was carried out for 42 min at 37°C in a gentleMACS Octo Dissociator (Miltenyi, 130-096-427). Prior to antibody staining, the cell suspension was filtered through a 50 μm nylon mesh and red blood cells were lysed in ACK buffer (150 mM NH_4_Cl, 10 mM KHCO_3_, 0.1 mM Na_2_EDTA). Gating of only viable cells was ensured by staining with Viability dye (Zombie NIR, 1:1000) for 15 min at room temperature in the dark, while nonspecific binding of antibodies was reduced by blocking the CD32/CD16 receptor for 20 min at 4°C (TruStain FcX (anti CD32/16 antibody), 1:50). The cells were then incubated with conjugated primary antibodies for 15 min at 4°C (anti-CD103, 1:100; anti-CD11b, 1:500; anti-CD11c, 1:200; anti-CD138, 1:200; anti-CD19, 1:800; anti-CD25, 1:100; anti-CD3, 1:50; anti-CD4, 1:1000; anti-CD45, 1:200; anti-CD8, 1:200; anti-CTLA4/CD152, 1:20; anti-F4/80, 1:100; anti-IgD, 1:100; anti-Ly6C, 1:1000; anti-Ly6G, 1:200; anti-MHCII, 1:200; anti-NK1.1, 1:100; anti-PD-1/CD279, 1:200; anti-CD38, 1:300; anti-CD44, 1:400; anti-CD80, 1:200; anti-TIM-3/CD366, 1:200). For intracellular staining, the cell suspensions were further fixed and permeabilized using a FOXP3/Transcription factor staining buffer set according to the manufacturer’s instructions. Antibodies targeting intracellular antigens (anti-FoxP3, 1:50; anti-TCF-1, 1:300) were incubated with the cells for 15 min at 4°C. After washing the samples, images were acquired with an Attune NxT flow cytometer (Invitrogen) and data were analyzed using FlowJo v10 software (ThreeStar). Representative gating strategy is reported in Figure S2A.

#### Platelet LAMP-1 exposure

After severing the tail of anesthetized mice, whole blood was collected into EDTA (6 mM). Blood was stimulated with agonists (collagen-related peptide 10 μg/mL and PAR-4 peptide 1 mmol/L) for 10 min and then incubated with LAMP-1 FITC antibody (5 μg/mL) for 30 min. Fluorescence (10,000 platelet events) was determined by flow cytometry (Gallios, Beckman Coulter).

#### Thrombin generation assay

Blood was collected into citrate (3.15%) from the abdominal aorta of anesthetized mice. Whole blood of individual mice was centrifuged to obtain PRP. Calibrated automated thrombogram (CAT) plate was filled with 20 μL of PRP reagent and 80 μL of PRP were added and incubated for 10 min. Then 20 μL of FluCa were added. CAT system was used to determine thrombin generation over time.

### Quantification and statistical analysis

Excel 6.0 and GraphPad Prism 9.5.0 were used for post-processing of the data. For statistical analysis, a Shapiro-Wilk normality test was applied before the statistical test. Depending on the Gaussian or non-Gaussian distribution of the data, an unpaired t-test (with Welch’s correction when needed) or a Mann-Whitney (for comparison of 2 groups) was used. Longitudinal analyses were performed by two-way ANOVA corrected with the Original FDR method of Benjamini and Hochberg. A Mantel-Cox test was employed for the bleeding time and a Fisher’s exact test for the classification of tumor volume. The numbers of replicates and independent experiments are indicated in the figure legends. Data information: ns *p* > 0.05; ∗*p* < 0.05; ∗∗*p* < 0.01; ∗∗∗*p* < 0.001; ∗∗∗∗*p* < 0.0001.
