# Supplementary material for: Depletion of all platelet integrins impacts hemostasis, thrombosis, and tumor metastasis
Source: iScience. 2025 Jul 31;28(9):113250. doi: 10.1016/j.isci.2025.113250 (PMC12396298; doi:10.1016/j.isci.2025.113250)
Supplement: Document S1. Figures S1 and S2 [file mmc1.pdf]

iScience, Volume *28*

Supplemental information

Depletion of all platelet integrins impacts hemostasis, thrombosis, and tumor metastasis

Emily Janus-Bell, Cristina Liboni, Alexandra Yakusheva, Vincent Mittelheisser, Clarisse Mouriaux, Catherine Bourdon, Louis Bochler, Vincent Hyenne, Maria Garcia-Leon, Olivier Lefebvre, Jacky G. Goetz, and Pierre H. Mangin

Figure S1

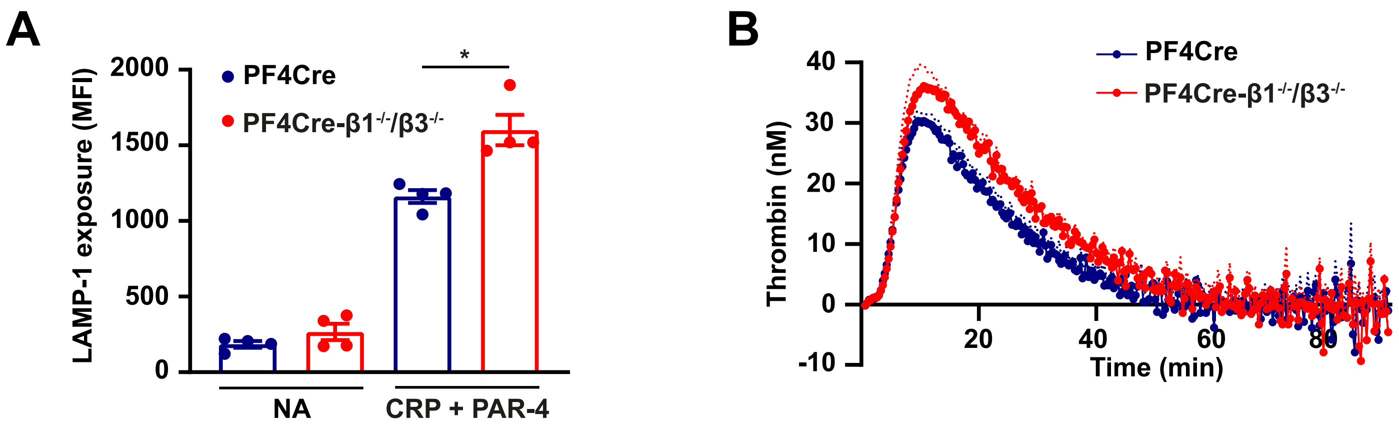
A
LAMP-1 exposure (MFI)
2000
1500
1000
500
0
PF4Cre
PF4Cre-β1-/-/β3-/-
*
NA
CRP + PAR-4
B
Thrombin (nM)
40
30
20
10
0
-10
20
40
60
80
Time (min)
PF4Cre
PF4Cre-β1-/-/β3-/-

Figure S2

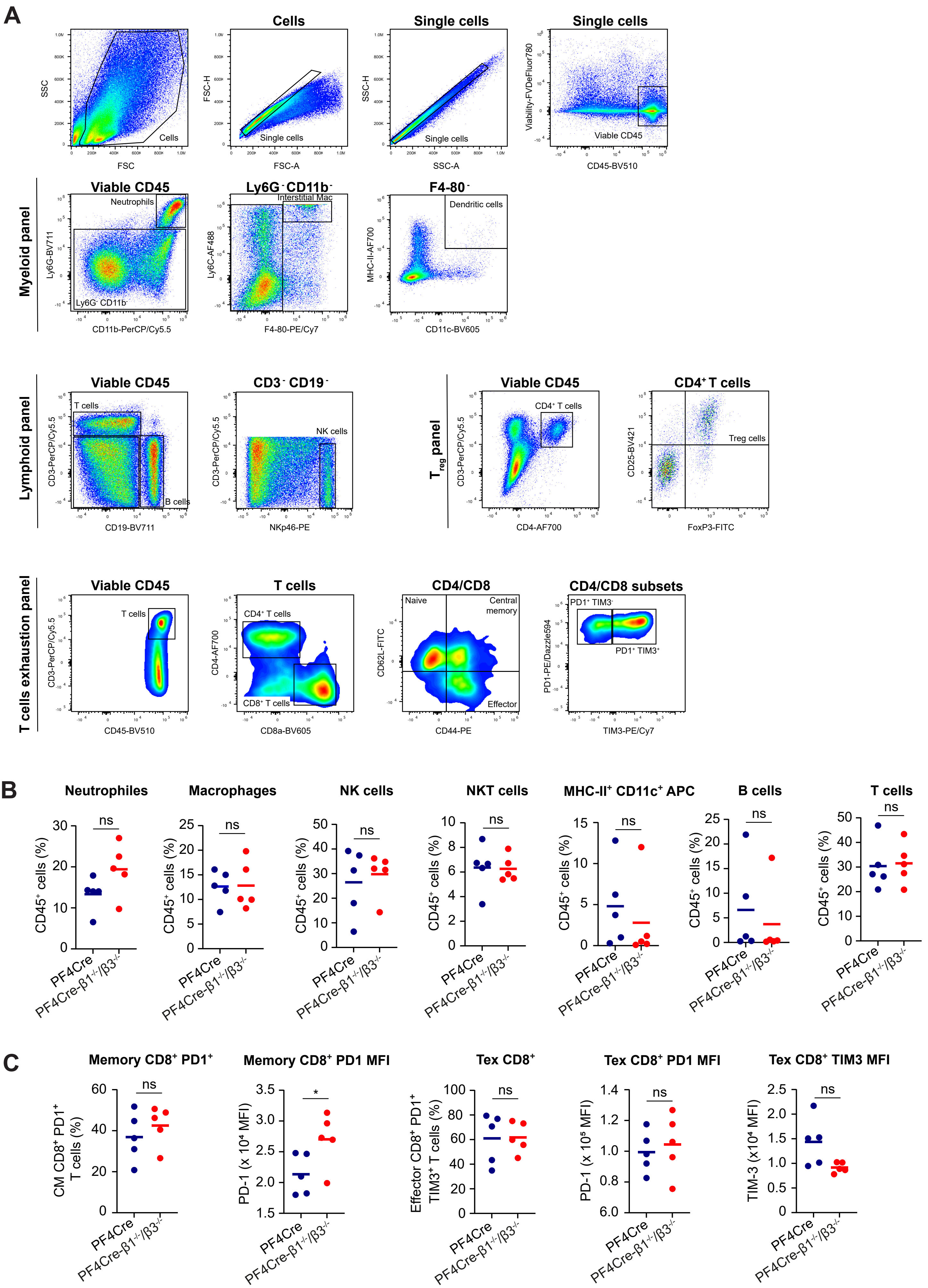

A
Cells
Single cells
Single cells
Viable CD45
Myeloid panel
Viable CD45
Ly6G- CD11b-
F4-80-
Neutrophils
Interstitial Mac
Dendritic cells
Lymphoid panel
Viable CD45
CD3- CD19-
T cells
NK cells
B cells
Treg panel
Viable CD45
CD4+ T cells
Treg cells
T cells exhaustion panel
Viable CD45
T cells
CD4/CD8
CD4/CD8 subsets
CD4+ T cells
CD8+ T cells
Naive
Central memory
Effector
PD1+ TIM3-
PD1+ TIM3+
B
Neutrophiles
Macrophages
NK cells
NKT cells
MHC-II+ CD11c+ APC
B cells
T cells
CD45+ cells (%)
ns
PF4Cre
PF4Cre-β1-/-/β3-/-
C
Memory CD8+ PD1+
Memory CD8+ PD1 MFI
Tex CD8+
Tex CD8+ PD1 MFI
Tex CD8+ TIM3 MFI
CM CD8+ PD1+ T cells (%)
PD-1 (x 10^4 MFI)
Effector CD8+ PD1+ TIM3+ T cells (%)
PD-1 (x 10^5 MFI)
TIM-3 (x10^4 MFI)
ns
*

Figure S1. Supplementary Figure relative to Figure 1. A. LAMP-1 expression using flow cytometry assay. LAMP-1 expression at the platelet surface is represented in non-activated and activated conditions (CRP + PAR-4 peptide) for PF4Cre (n=4) and PF4Cre-$\beta1^{-/-}/\beta3^{-/-}$ (n=4). Data are from 1 experiment and Mann-Whitney test was applied. **B.** Platelet-based thrombin generation assay. Thrombin generation over time from platelet-rich plasma using calibrated automated thrombogram (CAT) is represented for PF4Cre (n=3) and PF4Cre-$\beta1^{-/-}/\beta3^{-/-}$ (n=3). Data are from 2 experiments and were analyzed with a Mann-Whitney test. Data information: * $p < 0.05$.

Figure S2. Supplementary Figure relative to Figure 3. A. Tumor immunophenotyping gating strategy. From the top: single cell gating strategy, myeloid panel gating, lymphoid panel gating, Treg gating and T cell exhaustion gating. **B.** *Ex-vivo* tumor immunophenotyping by flow-cytometry. Percentages of immune cell populations within the defined gating are represented for PF4Cre (n=5) and PF4Cre-$\beta1^{-/-}/\beta3^{-/-}$ (n=6). Data are from 1 experiment and Mann-Whitney test was applied. **C.** Expression levels (mean fluorescence index) of PD1 and TIM3 molecules of memory $CD8^+$ (Central Memory-CM) and T exhausted cells (Tex) as measured by flow-cytometry for PF4Cre (n=5) and PF4Cre-$\beta1^{-/-}/\beta3^{-/-}$ (n=5). Data are from 1 experiment and Mann-Whitney test was applied. Data information: ns $p > 0.05$; * $p < 0.05$.
